# Mediating role of inflammatory markers in the association between dietary isorhamnetin consumption and CKD risk

**DOI:** 10.1097/MD.0000000000044703

**Published:** 2025-10-10

**Authors:** Chang Liu, Hanhan Kong, Guixia Li, Wujian Peng, Peijia Liu

**Affiliations:** a Department of Nephrology, Shenzhen Third People’s Hospital, The Second Affiliated Hospital of Southern University of Science and Technology, Shenzhen, Guangdong, China.

**Keywords:** albuminuria, chronic kidney disease, inflammation, isorhamnetin, mediation analysis, NHANES

## Abstract

The association between dietary isorhamnetin (ISO) intake and chronic kidney disease (CKD) risk remains unclear. Additionally, the role of inflammation as a potential mediator in this association warrants further investigation. This study aimed to explore the mediating role of inflammatory markers in the association between dietary ISO intake and CKD risk. This cross-sectional study used the data from the National Health and Nutrition Examination Survey 2007 to 2010 and 2017 to 2018 cycles. Dietary ISO intake was assessed through 24-hour dietary recalls. CKD was defined as an estimated glomerular filtration rate <60 mL/min/1.73 m^2^ and/or a urinary albumin-to-creatinine ratio ≥30 mg/g. Weighted multivariable logistic regression models and restricted cubic splines were employed to assess the association between ISO intake and CKD risk. Mediation analysis was performed to examine the potential role of inflammatory markers, including white blood cell count, neutrophil count (NEUT), red cell distribution width, systemic immune-inflammation index, and neutrophil-to-lymphocyte ratio. Higher dietary ISO (ln-transformed) intake was significantly associated with a lower risk of albuminuria (Q4 vs Q1: odds ratio = 0.669, 95% confidence interval: 0.515–0.870, *P* for trend = .021) but not with CKD risk or estimated glomerular filtration rate decline. Restricted cubic splines analysis revealed a significant negative linear correlation between ISO (ln-transformed) intake and albuminuria (*P* = .037). The mediation analysis indicated that the protective effect of ISO on albuminuria was partially explained by the reductions in NEUT (8.9%), red cell distribution width (6.6%), systemic immune-inflammation index (6.7%), neutrophil-to-lymphocyte ratio (6.7%), and white blood cell count (4.3%), with NEUT exhibiting the strongest indirect effect. While our findings provide epidemiological evidence that higher dietary ISO intake is associated with reduced albuminuria risk, with inflammatory markers mediating a modest proportion of this association, the limited effect sizes warrant cautious interpretation regarding potential anti-inflammatory mechanisms. Further longitudinal and interventional studies are warranted to confirm these findings and explore the therapeutic potential of ISO in preventing CKD.

## 1. Introduction

Chronic kidney disease (CKD) poses a significant global public health challenge, necessitating urgent attention. Estimates show that approximately 700 million individuals are affected by CKD worldwide, and this number continues to rise steadily.^[1]^ The prevalence of CKD is equally concerning in the United States. The data from the Centers for Disease Control and Prevention indicate that around 37 million Americans, or about 15% of the population, are affected by this condition.^[2]^ The management of CKD typically focuses on lifestyle changes, such as adopting a low-salt and low-protein diet, along with controlling risk factors such as proteinuria, hypertension, hyperglycemia, hyperuricemia, and dyslipidemia.^[3,4]^ However, despite these strategies, effective treatment options for CKD remain limited, thereby highlighting the need for discovering novel therapeutic interventions.

Isorhamnetin (ISO) is a bioactive flavonoid primarily derived from the fruits of *Hippophae rhamnoides* and the leaves of *Ginkgo biloba*. It exhibits a diverse range of pharmacological properties, including cardiovascular and cerebrovascular protection, as well as anti-inflammatory and antioxidant effects.^[5]^ These beneficial effects are primarily attributed to the ability of ISO to modulate several key signaling pathways, such as mitogen-activated protein kinase and phosphoinositide 3-kinase/protein kinase B, which are critically involved in inflammation, oxidative stress, and cellular homeostasis.^[5–9]^ Besides, ISO has demonstrated therapeutic effects in metabolic disorders including obesity, diabetes, and metabolic syndrome.^[9–11]^ Furthermore, ISO exhibits protective effects in the liver and lungs, which reinforces its role in mitigating diseases associated with chronic inflammation and oxidative damage.^[12–14]^ Notably, emerging evidence also supports the renoprotective effects of ISO, particularly in diabetic nephropathy, acute kidney injury, and renal fibrosis.^[8,15–17]^ Collectively, the anti-inflammatory, antioxidant, and antifibrotic properties of ISO, alongside its capacity to regulate critical signaling pathways, highlight its potential as a promising therapeutic agent for preventing and treating CKD.

Building on previous findings, we hypothesize that higher dietary intake of ISO is associated with a lower risk of CKD. To evaluate this hypothesis, we conducted a cross-sectional analysis using the data from the National Health and Nutrition Examination Survey (NHANES), a US population-based database. Given the pivotal role of inflammation in CKD pathogenesis, the causal mediation analysis was performed to assess how much inflammation mediates the association between dietary ISO intake and CKD risk.

## 2. Methods

### 2.1. Study design and study population

The NHANES database is a publicly accessible, nationally representative database that collects data biennially through a complex, multistage, stratified sampling design. It covers extensive information, including demographics, dietary intake, physical examinations, laboratory test results, and health-related survey responses. NHANES ensures the representativeness of the US population by applying specific sampling weights to adjust for oversampling and nonresponse biases.^[18]^

**Figure 1.**
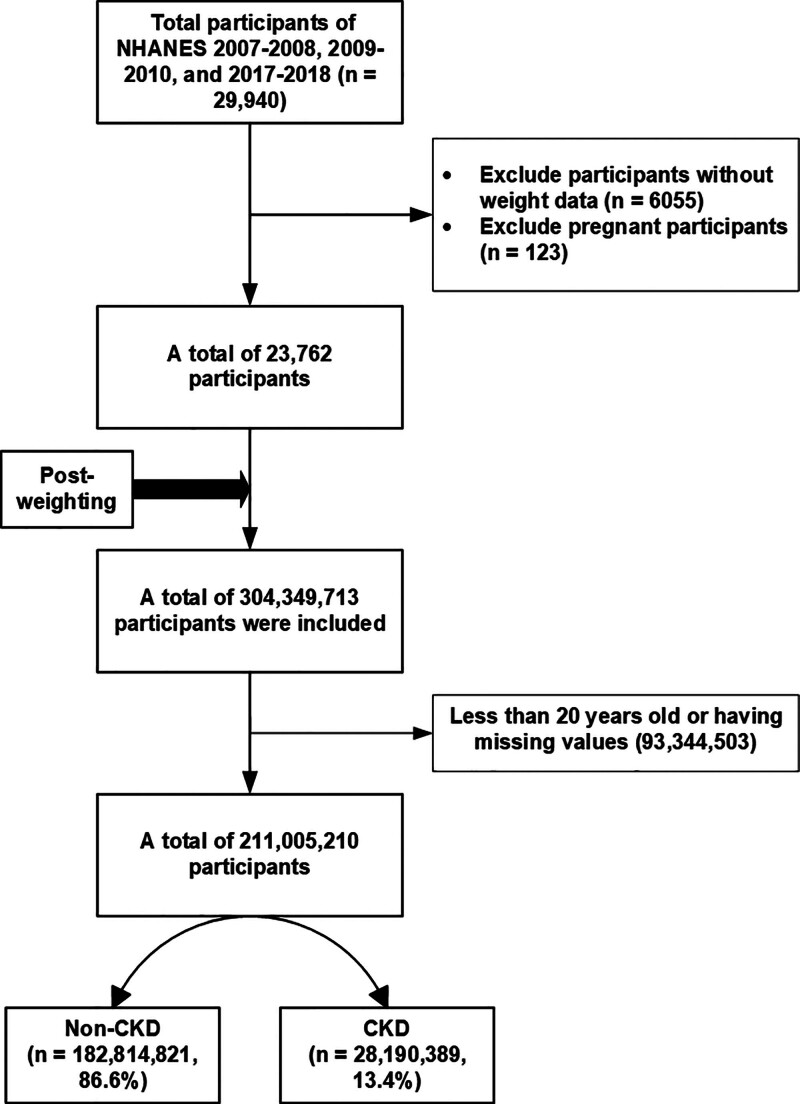
Flowchart of participants selection. CKD = chronic kidney disease, NHANES = National Health and Nutrition Examination Survey.

This study employed a cross-sectional design, integrating flavonoid intake data from the United States Department of Agriculture (USDA) Food Codes. The data from 3 NHANES cycles (2007–2008, 2009–2010, and 2017–2018) were included, with 29,940 participants. Initially, we excluded participants with missing weight data (n = 6055) and pregnant women (n = 123), resulting in a sample of 23,762 participants. After applying sampling weights, the dataset represented approximately 304,349,713 US individuals. Further exclusions were applied to ensure data completeness and relevance: individuals aged <20 years, those lacking estimated glomerular filtration rate (eGFR) measurements, those without albuminuria data, and those with no reported ISO intake were excluded. The final analytical sample comprised 211,005,210 individuals, as illustrated in Figure 1.

### 2.2. Consumption of ISO

The daily nutrient intake from food sources was estimated using a standardized methodology developed by the USDA.^[19–21]^ Trained interviewers used the automated multiple-pass method to conduct 24-hour dietary recalls, with the recall on Day 1 completed in-person at the NHANES Mobile Examination Center and that on Day 2 conducted via telephone within 3 to 10 days.^[22]^ ISO intake was quantified by multiplying the ISO content of each food item by its reported consumption frequency, as recorded in the food frequency questionnaire. The food codes from the Food and Nutrient Database for Dietary Studies (FNDDS) were used to identify and determine the content of ISO-containing foods. Specifically, FNDDS version 4.1 was applied for the NHANES 2007 to 2008 cycle, whereas FNDDS version 5.0 was used for the 2009 to 2010 and 2017 to 2018 cycles.^[19–21]^ The total consumption from both Day 1 and Day 2 dietary recalls was averaged and used as the primary exposure variable to assess individual ISO intake.

### 2.3. Outcome assessment

The urinary albumin levels were measured using a solid-phase fluorescence immunoassay,^[23]^ whereas the serum and urine creatinine concentrations were determined using the Jaffe method.^[24]^ The eGFR was calculated using the 2021 Chronic Kidney Disease Epidemiology Collaboration (CKD-EPI) equation based on serum creatinine levels.^[25]^ CKD was defined as an eGFR of <60 mL/min/1.73 m^2^ and/or a urinary albumin-to-creatinine ratio >30 mg/g, in accordance with the Kidney Disease Outcomes Quality Initiative guidelines.^[3,4,26]^ The primary outcome was CKD occurrence, whereas secondary endpoints included proteinuria (urinary albumin-to-creatinine ratio >30 mg/g) and a decreased eGFR (eGFR < 60 mL/min/1.73 m^2^).

### 2.4. Inflammatory biomarkers

This study incorporated multiple inflammatory biomarkers to assess systemic inflammation and immune responses. C-reactive protein (CRP) and high-sensitivity CRP (hsCRP) serve as the key markers of acute-phase inflammation, with hsCRP offering a more sensitive measure of low-grade systemic inflammation.^[27,28]^ White blood cell count (WBC) reflects overall immune activity, whereas its subtypes (neutrophil count [NEUT], lymphocyte count, and monocyte count) play distinct roles in host defense, immune modulation, and chronic inflammation. Moreover, red cell distribution width (RDW), a marker of erythrocyte size variability, has been increasingly recognized as an indicator of systemic inflammation and oxidative stress.^[29]^ Further, composite indices, including the systemic immune-inflammation index (SII), neutrophil-to-lymphocyte ratio (NLR), platelet-to-lymphocyte ratio, and lymphocyte-to-monocyte ratio, integrate hematological parameters to provide a more comprehensive assessment of immune and inflammatory status.^[30–32]^ Among these, SII is calculated as (platelet count × neutrophil count)/lymphocyte count, serving as a valuable marker of systemic inflammation. Collectively, these inflammatory markers offer critical insights into inflammatory processes, thereby helping evaluate their role in disease risk and progression.

### 2.5. Covariates

The covariates incorporated in this study encompassed demographic characteristics, lifestyle factors, and clinical parameters. The demographic variables included age (years), sex (male or female), and race/ethnicity (Mexican American, non-Hispanic White, non-Hispanic Black, and others, including multiracial individuals). Educational attainment was categorized as middle school or below versus high school or above, whereas marital status was classified as married versus others. Economic status was assessed using the poverty income ratio (PIR), with a PIR < 1 indicating poverty and a PIR ≥ 1 representing nonpoverty. The lifestyle-related variables included smoking status, physical activity, and dietary energy intake. Smoking status was categorized as never smokers (consumed fewer than 100 cigarettes in their lifetime), former smokers (consumed at least 100 cigarettes but had quit), and current smokers (consumed at least 100 cigarettes and continued smoking, either regularly or occasionally). Physical activity was assessed using the metabolic equivalent of task minutes per week and dichotomized at 600 metabolic equivalent of task minutes/week.^[33]^ The dietary energy intake was calculated as the average total kilocalories consumed over two 24-hour dietary recalls. Anthropometric and clinical parameters included body mass index (BMI), blood pressure, lipid profile, blood glucose levels, and serum uric acid concentration. BMI was computed as weight in kilograms divided by the square of height in meters (kg/m^2^). Blood pressure measurements were recorded as the average of 3 readings, with hypertension defined as a systolic blood pressure ≥140 mm Hg, diastolic blood pressure ≥90 mm Hg, use of antihypertensive medications, or a clinical diagnosis of hypertension.^[34]^ Dyslipidemia was identified based on at least one of the following criteria: triglycerides >150 mg/dL, total cholesterol >200 mg/dL, low-density lipoprotein cholesterol >130 mg/dL, high-density lipoprotein cholesterol <40 mg/dL in men or <50 mg/dL in women, or current use of lipid-lowering therapy.^[35]^ Diabetes was defined by the presence of one or more of the following conditions: glycated hemoglobin >6.5%, fasting plasma glucose >7.0 mmol/L, random plasma glucose >11.1 mmol/L, 2-hour postprandial glucose >11.1 mmol/L, use of antidiabetic medication, or a clinical diagnosis of diabetes.^[36]^ Hyperuricemia was defined as serum uric acid levels exceeding 360 µmol/L in women or 420 µmol/L in men.^[37]^

### 2.6. Statistical analyses

The NHANES dataset was collected using a complex, multistage stratified sampling approach. The data were appropriately weighted before statistical analysis to ensure that the results represented the broader US population. The continuous variables were summarized as means with 95% confidence intervals (CIs) and compared between CKD and non-CKD groups using the Wilcoxon rank-sum test. The categorical variables were expressed as percentages with corresponding 95% CIs, and the differences between groups were evaluated using the chi-square test. Dietary ISO intake was natural log (ln)-transformed to approximate normality for continuous variable analyses. The ln-transformed values were further categorized into quartiles (Q1–Q4) for categorical analyses. The weighted multivariable logistic regression models were employed, examining CKD risk, eGFR decline, and proteinuria, to evaluate the relationship between ln-transformed ISO intake and renal outcomes. Restricted cubic splines (RCS) were applied to assess potential nonlinear dose-response relationships between ln-transformed ISO intake and CKD risk. Stratified analyses were conducted to explore whether the association between ln-transformed ISO intake and albuminuria risk varied across different subgroups. The key inflammatory mediators were identified using weighted multiple linear regression models to examine the associations between ln-transformed ISO intake and inflammatory biomarkers. The mediation analysis was performed to assess the indirect effects of inflammatory markers on the association between ISO intake and proteinuria risk, with CIs estimated using nonparametric bootstrapping (n = 1000). All statistical analyses were conducted using R version 4.3.0 (University of Auckland, Auckland, New Zealand), with statistical significance set at a 2-tailed *P* < .05.

## 3. Results

### 3.1. Characteristics of the population

Table 1 presents the baseline characteristics of the study population, categorized by CKD status. Of the study participants, 13.4% were included in the CKD group and the remaining 86.6% in the non-CKD group. Compared with non-CKD participants, patients with CKD were generally older and exhibited a higher prevalence of hypertension, diabetes, hyperlipidemia, and hyperuricemia. Moreover, they demonstrated unfavorable metabolic and lifestyle profiles, including lower physical activity levels and reduced dietary energy intake. Patients with CKD had significantly lower dietary ISO intake than their non-CKD counterparts, highlighting the need for further exploring its potential protective role in CKD prevention.

**Table 1:** Baseline characteristics by CKD status in US population.

Variables	Total population (100%)	Non-CKD group (86.6%)	CKD group (13.4%)	*P* value
Age (yr)	47.7 (47.0–48.3)	45.8 (45.1–46.4)	60.1 (59.0–61.2)	**<.001**
Sex (%) (95% CI)				**.001**
Female	52.2 (48.9–55.5)	51.5 (50.3–52.6)	56.9 (53.8–60.0)	
Male	47.8 (44.8–50.8)	48.5 (47.4–49.7)	43.1 (40.0–46.2)	
Ethnicity (%) (95% CI)				**<.001**
Black	10.9 (9.3–12.5)	10.3 (8.5–12.0)	15.1 (12.5–17.7)	
White	67.7 (60.9–74.5)	67.8 (64.1–71.5)	66.9 (62.8–71.0)	
Mexican	8.6 (6.8–10.4)	8.7 (6.6–10.7)	8.1 (6.3–9.9)	
Others	12.8 (11.1–14.5)	13.2 (11.3–15.1)	10.0 (7.8–12.2)	
Education levels (%) (95% CI)				.2
High school or above	55.3 (51.4–59.3)	55.1 (52.8–57.4)	57.3 (54.0–60.6)	
Middle school or below	44.6 (41.2–47.9)	44.9 (42.6–47.2)	42.7 (39.4–46.0)	
Height (cm)	168.5 (168.3–168.8)	168.9 (168.6–169.2)	166.1 (165.5–166.6)	**<.001**
Weight (kg)	83.1 (82.4–83.8)	82.8 (82.1–83.5)	85.4 (83.5–87.3)	**.01**
BMI (kg/m^2^)	29.2 (28.9–29.4)	28.9 (28.7–29.2)	30.7 (30.1–31.4)	**<.001**
Overweight (%) (95% CI)				**<.001**
No	28.7 (26.3–31.1)	29.7 (28.0–31.5)	22.8 (20.1–25.6)	
Yes	70.8 (66.5–75.1)	70.3 (68.5–72.0)	77.2 (74.4–79.9)	
SBP (mm Hg)	121.6 (121.0–122.2)	120.0 (119.5–120.6)	131.8 (130.3–133.4)	**<.001**
DBP (mm Hg)	71.1 (70.4–71.7)	71.3 (70.6–71.9)	70.0 (69.0–70.9)	**.01**
PIR (%) (95% CI)				.1
≥1	80.0 (74.6–85.5)	86.5 (85.1–87.9)	85.1 (83.2–87.0)	
<1	12.7 (11.6–13.8)	13.5 (12.1–14.9)	14.9 (13.0–16.8)	
Energy intake (kcal)	2135 (2106–2164)	2170 (2139–2202)	1909 (1851–1967)	**<.001**
Physical activity status (%) (95% CI)				**<.001**
<600 MET min/week	12.8 (11.5–14.1)	15.4 (14.0–16.7)	24.8 (21.7–27.9)	
≥600 MET min/week	65.6 (61.3–69.9)	84.6 (83.3–86.0)	75.2 (72.1–78.3)	
Smoking status (%) (95% CI)				**<.001**
Former	25.1 (23.1–27.1)	24.0 (22.5–25.5)	32.5 (30.2–34.9)	
Never	55.7 (52.1–59.2)	56.4 (54.3–58.6)	50.7 (47.4–54.1)	
Now	19.2 (17.2–21.2)	19.6 (18.2–21.0)	16.7 (14.1–19.4)	
Hyperlipidemia (%) (95% CI)				**<.001**
No	28.4 (26.1–30.7)	29.9 (28.1–31.8)	18.4 (16.0–20.9)	
Yes	71.6 (67.0–76.2)	70.1 (68.2–71.9)	81.6 (79.1–84.0)	
Hypertension (%) (95% CI)				**<.001**
No	62.7 (59.1–66.2)	67.5 (65.6–69.3)	31.4 (28.4–34.4)	
Yes	37.3 (34.1–40.5)	32.5 (30.7–34.4)	68.6 (65.6–71.6)	
Diabetes (%) (95% CI)				
No	85.6 (80.3–90.8)	89.3 (88.5–90.1)	62.6 (59.9–65.3)	**<.001**
Yes	14.3 (13.1–15.4)	10.7 (9.9–11.5)	37.4 (34.7–40.1)	
Hyperuricemia (%) (95% CI)				
No	81.2 (76.1–86.2)	83.8 (82.7–84.9)	65.9 (63.3–68.6)	**<.001**
Yes	18.4 (17.1–19.7)	16.2 (15.1–17.3)	34.1 (31.4–36.7)	
eGFR (mL/min/1.73 m^2^)	95.8 (95.0–96.7)	98.9 (98.0–99.8)	75.4 (73.7–77.1)	**<.001**
UACR (mg/g)	34.0 (28.2–39.8)	7.5 (7.3–7.7)	208.5 (166.9–250.2)	**<.001**
Isorhamnetin (mg)	0.80 (0.77–0.82)	0.81 (0.78–0.84)	0.70 (0.65–0.74)	**<.001**

Data are presented as mean for continuous variables or proportions for categorical variables with adjusted 95% confidence interval. Bold values indicate statistically significant (*P* < .05).

BMI = body mass index, CI = confidence interval, CKD = chronic kidney disease, DBP = diastolic blood pressure, eGFR = estimated glomerular filtration rate, MET = metabolic equivalent of task, PIR = poverty income ratio, SBP = systolic blood pressure, UACR = urinary albumin-to-creatinine ratio.

### 3.2. Association between dietary ISO intake and CKD

Table 2 presents the associations between ln-transformed dietary ISO intake and CKD risk, decreased eGFR, and albuminuria, adjusted for multiple covariates in Model 3. Compared with Q1, higher ISO (ln-transformed) intake was associated with lower odds of CKD, with the following odds ratios (ORs) and 95% CIs: Q2 (OR = 0.714, 95% CI: 0.550–0.926), Q3 (OR = 0.743, 95% CI: 0.580–0.952), and Q4 (OR = 0.740, 95% CI: 0.569–0.964). However, the overall trend was not statistically significant (*P* = .1).

**Table 2:** Association of dietary isorhamnetin (ln-transformed) consumption with CKD, decreased eGFR, and albuminuria in US population.

Outcomes	Total isorhamnetin (ln-transformed)(mg per day)				*P* value for trend
	OR (95% CI)				
	Q1	Q2	Q3	Q4	
	[0–0.039]	[0.039–0.259]	[0.259–0.579]	[0.579–2.006]	
CKD					
Model 1	ref	0.817 (0.666–1.002)	0.829 (0.674–1.021)	**0.684 (0.555–0.842**)	
Model 2	ref	**0.778 (0.618–0.979**)	**0.760 (0.621–0.928**)	**0.641 (0.518–0.794**)	
Model 3	ref	**0.714 (0.550–0.926**)	**0.743 (0.580–0.952**)	**0.740 (0.569–0.964**)	.1
Decreased eGFR					
Model 1	ref	1.025 (0.759–1.386)	0.880 (0.580–1.336)	0.749 (0.549–1.023)	
Model 2	ref	0.966 (0.666–1.399)	0.786 (0.500–1.235)	0.737 (0.514–1.057)	
Model 3	ref	1.012 (0.635–1.615)	0.871 (0.470–1.614)	0.868 (0.512–1.469)	.451
Albuminuria					
Model 1	ref	**0.740 (0.598–0.916**)	**0.757 (0.632–0.906**)	**0.625 (0.509–0.767**)	
Model 2	ref	**0.726 (0.582–0.906**)	**0.721 (0.607–0.858**)	**0.603 (0.496–0.732**)	
Model 3	ref	**0.624 (0.441–0.882**)	**0.667 (0.484–0.920**)	**0.669 (0.515–0.870**)	**.021**

Model 1 did not include any covariate. Model 2 was adjusted for age and sex. Model 3 was adjusted for age, sex, body mass index, ethnicity, smoke status, energy intake, physical activity status, hyperuricemia, hyperlipidemia, hypertension, and diabetes mellitus. Bold values indicate statistically significant (*P* < .05).

CI = confidence interval, CKD = chronic kidney disease, eGFR = estimated glomerular filtration rate, OR = odds ratio, Q1 = quartile 1, Q2 = quartile 2, Q3 = quartile 3, Q4 = quartile 4.

**Figure 2.**
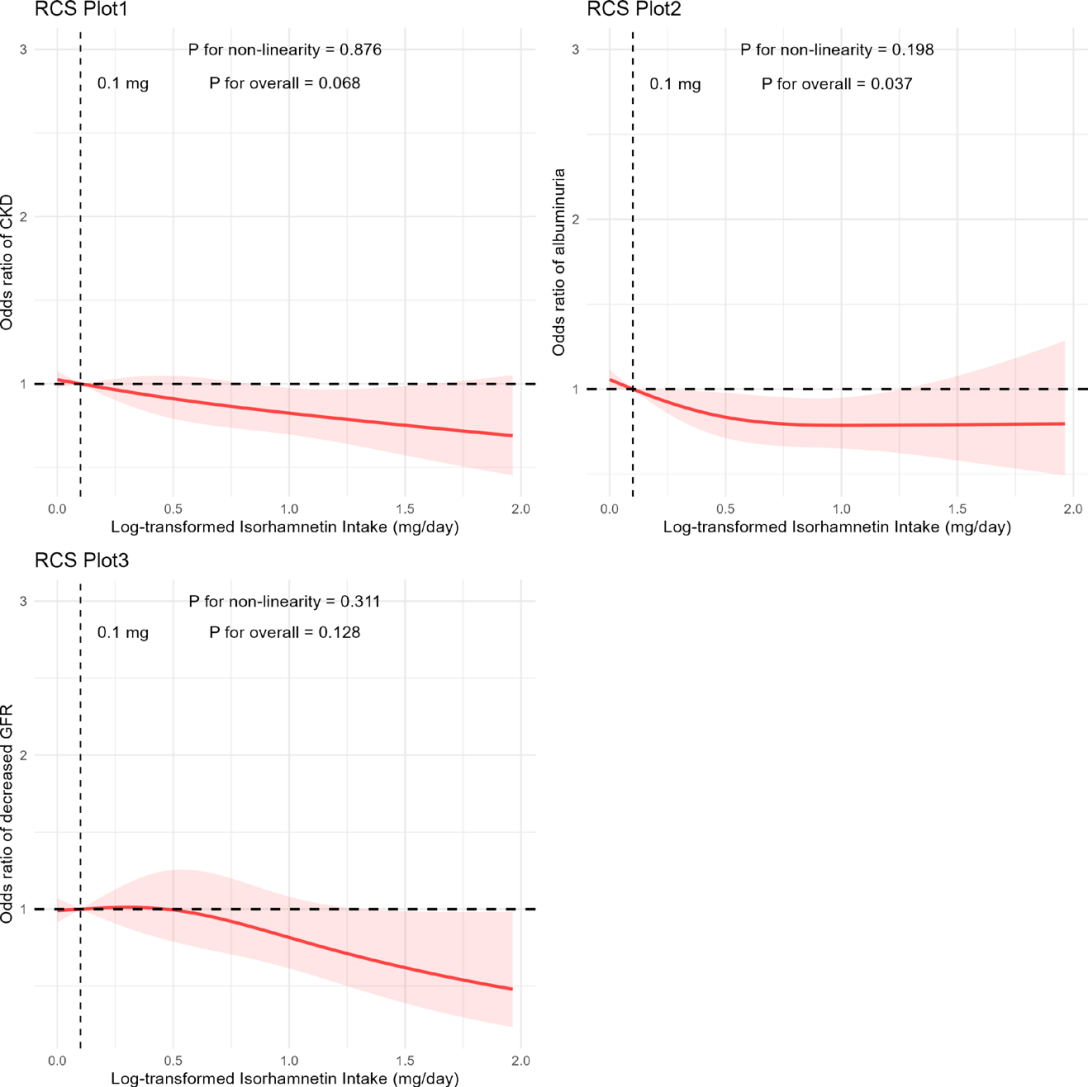
Nonlinear relationship between isorhamnetin (ln-transformed) intake and CKD, decreased eGFR, and albuminuria using RCS. Data are presented as odds ratios of adverse renal outcomes (*y*-axis) and isorhamnetin intake (ln-transformed) (mg/day) after adjustment for age, sex, body mass index, ethnicity, smoking status, energy intake, physical activity, hyperuricemia, hyperlipidemia, hypertension, and diabetes mellitus. CKD = chronic kidney disease, eGFR = estimated glomerular filtration rate, RCS = restricted cubic spline.

No significant association was observed between ISO (ln-transformed) intake and eGFR decline, with ORs as follows: Q2 (OR = 1.012, 95% CI: 0.635–1.615), Q3 (OR = 0.871, 95% CI: 0.470–1.614), and Q4 (OR = 0.868, 95% CI: 0.512–1.469). On the contrary, higher ISO intake was significantly associated with lower odds of albuminuria, with ORs as follows: Q2 (OR = 0.624, 95% CI: 0.441–0.882), Q3 (OR = 0.667, 95% CI: 0.484–0.920), and Q4 (OR = 0.669, 95% CI: 0.515–0.870), demonstrating a statistically significant trend (*P* = .021). As illustrated in Figure 2, the RCS analysis revealed a negative linear correlation between ln-transformed ISO consumption and albuminuria risk (*P* for overall = 0.037). However, no significant nonlinear associations were detected between ISO intake and CKD, decreased eGFR, or albuminuria.

### 3.3. Stratified analysis of the association between dietary ISO intake and albuminuria risk

As shown in Table 3, after adjusting for multiple variables, the ORs for albuminuria risk in the highest quartile (Q4) compared with the lowest quartile (Q1), with 95% CIs, were as follows: 0.567 (0.390–0.824) for White individuals, 0.681 (0.475–0.976) for those with BMI ≥ 25 kg/m^2^, 0.620 (0.442–0.870) for individuals with hyperlipidemia, 0.416 (0.262–0.660) for those with diabetes, and 0.615 (0.472–0.801) for those with non-hyperuricemia. The trend tests for these subgroups revealed *P* values above .05, except for patients with diabetes (*P* < .001) and those with non-hyperuricemia (*P* = .003). The interaction analysis showed that the *P*-values for the interaction tests were above 0.05 for all subgroups, except for the diabetes subgroup.

**Table 3:** Stratified analyses by potential modifiers of the association between dietary isorhamnetin (ln-transformed) intake and albuminuria risk in US population.

Subgroup category	OR (95% CI)				*P* value for trend	*P* value for interaction
	Q1	Q2	Q3	Q4		
Sex						.912
Female	ref	0.665 (0.385–1.148)	0.663 (0.415–1.060)	0.744 (0.462–1.197)	.366	
Male	ref	0.569 (0.348–0.931)	0.671 (0.389–1.156)	0.597 (0.374–0.955)	.095	
Age (yr)						.882
20–39	ref	0.450 (0.202– 0.999)	0.781 (0.388–1.574)	0.731 (0.439–1.218)	.092	
40–59		0.584 (0.290–1.174)	0.587 (0.309–1.116)	0.577 (0.293–1.137)	.157	
60–	ref	0.770 (0.444–1.337)	0.639 (0.349–1.171)	0.669 (0.445–1.005)	.857	
Ethnicity						.473
Black	ref	0.589 (0.292–1.190)	0.678 (0.340–1.353)	0.713 (0.371–1.370)	.449	
White	ref	0.602 (0.374–0.969)	0.604 (0.375–0.972)	0.567 (0.390–0.824)	**.011**	
Mexican	ref	1.459 (0.633– 3.366)	1.713 (0.658–4.461)	1.305 (0.586– 2.907)	.831	
Others	ref	0.507 (0.239– 1.076)	0.715 (0.348–1.469)	0.903 (0.482– 1.692)	.589	
BMI (kg/m^2^)						.609
≥25	ref	0.649 (0.438–0.962)	0.625 (0.397–0.982)	0.681 (0.475–0.976)	.085	
<25	ref	0.530 (0.256–1.097)	0.786 (0.462–1.336)	0.572 (0.317–1.033)	.202	
Smoking status						.985
Never	ref	0.603 (0.390–0.931)	0.682 (0.430–1.082)	0.709 (0.460–1.092)	.325	
Former	ref	0.698 (0.379–1.285)	0.590 (0.305–1.144)	0.625 (0.407–0.960)	.062	
Now	ref	0.549 (0.251–1.203)	0.681 (0.403–1.149)	0.577 (0.276–1.207)	.211	
Hyperlipidemia						.153
Yes	ref	0.553 (0.384–0.797)	0.543 (0.350–0.842)	0.620 (0.442–0.870)	**.043**	
No	ref	0.905 (0.422–1.943)	1.266 (0.625–2.563)	0.834 (0.404–1.718)	.717	
Hypertension						.054
Yes	ref	0.642 (0.390–1.057)	0.474 (0.287–0.781)	0.652 (0.419–1.013)	.112	
No	ref	0.570 (0.355–0.913)	0.919 (0.609–1.386)	0.667 (0.467–0.954)	.261	
Diabetes mellitus						<.001
Yes	ref	0.663 (0.423–1.039)	0.663 (0.423–1.039)	0.798 (0.554–1.150)	.62	
No	ref	0.509 (0.269–0.961)	0.509 (0.269–0.961)	0.416 (0.262–0.660)	**<.001**	
Hyperuricemia						.767
Yes	ref	0.606 (0.276–1.333)	0.809 (0.398–1.646)	0.872 (0.425–1.790)	.954	
No	ref	0.609 (0.415–0.894)	0.629 (0.452–0.877)	0.615 (0.472–0.801)	**.003**	

The model was adjusted, if not stratified, for age, sex, body mass index, ethnicity, smoke status, energy intake, physical activity status, hyperuricemia, hyperlipidemia, hypertension, and diabetes mellitus. Bold values indicate statistically significant (*P* < .05).

BMI = body mass index, CI = confidence interval, OR = odds ratio, Q1 = quartile 1, Q2 = quartile 2, Q3 = quartile 3, Q4 = quartile 4.

### 3.4. Association between dietary ISO intake and Inflammatory markers

As shown in Table 4, higher dietary ISO intake was significantly associated with lower levels of several inflammatory markers. In the continuous variable analysis, ISO intake exhibited significant negative correlations with WBC (*β* = −0.212, 95% CI: −0.386 to −0.037, *P* = .019), NEUT (*β* = −0.156, 95% CI: −0.278 to −0.034, *P* = .014), RDW (*β* = −0.114, 95% CI: −0.177 to −0.051, *P* < .001), SII (*β* = −29.912, 95% CI: −58.215 to −1.610, *P* = .039), and NLR (*β* = −0.100, 95% CI: −0.195 to−0.005, *P* = .039), suggesting the association of increased ISO intake with reduced systemic inflammation. Quartile-based comparisons further supported these findings, as participants in Q4 (highest ISO intake) had significantly lower RDW compared with those in Q1 (reference), with *β* = −0.179 (95% CI: −0.276 to −0.082, *P* < .001), whereas those in Q3 also showed a significant reduction in RDW (*β* = −0.150, 95% CI: −0.253 to −0.048, *P* = .005). However, no significant associations were found between ISO intake and CRP, hsCRP, monocyte count, lymphocyte count, platelet-to-lymphocyte ratio, or lymphocyte-to-monocyte ratio in either the continuous or categorical analyses.

**Table 4:** Multiple linear regression of dietary isorhamnetin (ln-transformed) intake and inflammatory markers in adults based on NHANES 2007 to 2010 and 2017 to 2018.

Continuous isorhamnetin (ln-transformed) intakes			Category of isorhamnetin (ln-transformed) intakes						
	*β* (95% CI)	*P* value	Q1	Q2	*P* value	Q3	*P* value	Q4	*P* value
CRP	−0.039 (−0.087, 0.010)	.111	ref	−0.003 (−0.079, 0.074)	.937	−0.065 (−0.146, 0.016)	.107	−0.024 (−0.131, 0.084)	.645
hsCRP	−0.364 (−1.634, 0.906)	.551	ref	0.396 (−0.737, 1.530)	.468	−0.715 (−1.510, 0.079)	.074	−0.481 (−1.615, 0.653)	.380
WBC	**−0.212 (−0.386, −0.037**)	**.019**	ref	0.22 (−0.049, 0.489)	.106	−0.031 (−0.237, 0.174)	.758	−0.073 (−0.271, 0.124)	.453
NEUT	**−0.156 (−0.278, −0.034**)	**.014**	ref	0.078 (−0.082, 0.237)	.328	−0.008 (−0.157, 0.142)	.918	−0.085 (−0.248, 0.077)	.293
LYM	−0.048 (−0.168, 0.072)	.420	ref	0.135 (−0.046, 0.315)	.139	−0.007 (−0.089, 0.075)	.860	0.019 (−0.060, 0.098)	.633
MONO	−0.004 (−0.019, 0.011)	.583	ref	0.011 (−0.006, 0.027)	.211	−0.011 (−0.030, 0.007)	.225	0.001 (−0.018, 0.020)	.896
RDW	**−0.114 (−0.177, −0.051**)	**<.001**	ref	−0.101 (−0.218, 0.015)	.085	−0.15 (−0.253, −0.048)	**.005**	−0.179 (−0.276, −0.082)	**<.001**
SII	**−29.912 (−58.215, −1.610**)	**.039**	ref	−3.778 (−39.740, 32.183)	.832	−6.77 (−40.325, 26.785)	.683	−31.965 (−70.993, 7.062)	.105
NLR	**−0.1 (−0.195, −0.005**)	**.039**	ref	−0.017 (−0.138, 0.104)	.776	−0.019 (−0.129, 0.091)	.730	−0.1 (−0.224, 0.023)	.108
PLR	−2.825 (−6.763, 1.113)	.154	ref	−4.234 (−9.839, 1.372)	.133	−2.211 (−8.029, 3.608)	.444	−5.331 (−11.055, 0.394)	.067
LMR	−0.011 (−0.146, 0.123)	.867	ref	0.139 (−0.067, 0.344)	.179	0.152 (−0.008, 0.313)	.062	0.079 (−0.055, 0.212)	.237

Model was adjusted for age, sex, body mass index, ethnicity, smoke status, energy intake, physical activity status, hyperuricemia, hyperlipidemia, hypertension, and diabetes mellitus. Bold values indicate statistically significant (*P* < .05).

CI = confidence interval, CRP = C-reactive protein, hsCRP = high-sensitivity C-reactive protein, LMR = lymphocyte-to-monocyte ratio, LYM = lymphocyte count, MONO = monocyte count, NEUT = neutrophil count, NHANES = National Health and Nutrition Examination Survey, NLR = neutrophil-to-lymphocyte ratio, PLR = platelet-to-lymphocyte ratio, Q1 = quartile 1, Q2 = quartile 2, Q3 = quartile 3, Q4 = quartile 4, RDW = red cell distribution width, SII = systemic immune-inflammation index, WBC = white blood cell count.

### 3.5. Mediation effects of inflammatory markers on dietary ISO intake and albuminuria

As depicted in Table 5, among the inflammatory markers examined, NEUT exhibited the strongest mediation effect, accounting for 8.9% of the total effects (*β*_indirect_ = −0.0201, 95% CI: −0.0404 to −0.0040). RDW mediated 6.6% (*β*_indirect_ = −0.0149, 95% CI: −0.0303 to −0.0043), whereas SII and NLR each mediated 6.7% of the association (*β*_indirect_ = −0.0158, 95% CI: −0.0321 to −0.0038 and *β*_indirect_ = −0.0157, 95% CI: −0.0311 to −0.0037, respectively). WBC had the lowest mediation proportion at 4.3% (*β*_indirect_ = −0.0095, 95% CI: −0.0247 to −0.0017).

**Table 5:** The mediation effects of inflammatory markers on the association of dietary isorhamnetin (ln-transformed) intake with the albuminuria risk in US adults.

Mediators	Total effect, *β*	Direct effects, *β*	Indirect effects, *β* (95% CI)	Mediated proportion (%)
WBC	−0.2229	−0.2134	−0.0095 (−0.0247, −0.0017)	4.3
NEUT	−0.2251	−0.2050	−0.0201 (−0.0404, −0.0040)	8.9
RDW	−0.2251	−0.2102	−0.0149 (−0.0303, −0.0043)	6.6
SII	−0.2380	−0.2221	−0.0158 (−0.0321, −0.0038)	6.7
NLR	−0.2338	−0.2181	−0.0157 (−0.0311, −0.0037)	6.7

Model was adjusted for age, sex, body mass index, ethnicity, smoke status, energy intake, physical activity status, hyperuricemia, hyperlipidemia, hypertension, and diabetes mellitus.

CI = confidence interval, NEUT = neutrophil count, NLR = neutrophil-to-lymphocyte ratio, RDW = red cell distribution width, SII = systemic immune-inflammation index, WBC = white blood cell count.

## 4. Discussion

This nationally representative study showed a significant association of higher dietary ISO intake with a lower risk of albuminuria and overall CKD, but no significant association with eGFR decline. The reduction in CKD risk was primarily driven by decreased albuminuria. This protective effect was partially mediated by the reductions in inflammatory markers, particularly WBC, NEUT, RDW, SII, and NLR. The association was more pronounced among patients with diabetes and those without hyperuricemia, highlighting potential subgroup-specific benefits. Overall, these findings provide new insights into the complex interplay between dietary ISO intake, systemic inflammation, and renal health, suggesting that ISO may exert renoprotective effects through its anti-inflammatory properties, particularly in mitigating albuminuria risk.

Given the limited epidemiological data on the relationship between dietary ISO intake and kidney function, our findings enhance the understanding of the potential renoprotective effects of ISO. Previous studies have highlighted the beneficial role of flavonoids in maintaining renal health, which is primarily attributed to their anti-inflammatory and antioxidant properties.^[38]^ Some evidence suggests an association of higher flavonoid intake with a lower risk of adverse renal outcomes.^[21,39]^ Additionally, soy protein rich in flavonoids has been found to effectively lower serum creatinine, serum phosphate, urinary protein, and CRP levels in non-dialysis patients with CKD.^[40]^ Jiang et al further reported a potential association between higher flavonoid intake and reduced albuminuria risk, with CRP partially mediating this relationship.^[41]^ However, these studies have not specifically identified the flavonoid compounds playing a key role in renal protection. Beyond their direct effects on kidney function, flavanols (a subclass of flavonoids) have been widely recognized for enhancing endothelial function across various populations, including patients with diabetes, end-stage renal disease, and even those without preexisting conditions.^[42–44]^ Considering the contribution of endothelial dysfunction to renal impairment, flavonoid-mediated vascular improvements may confer indirect nephroprotective benefits. This hypothesis is further supported by the anti-inflammatory and antioxidant properties of flavonols; these flavonols mitigate oxidative stress and inflammation, which are the key drivers of CKD progression.^[45]^ Given the scarcity of data on the relationship between dietary ISO intake and CKD risk, our study enhances the understanding of this association and sheds light on the potential mediating role of inflammatory markers.

The inverse association between dietary ISO intake and albuminuria may be attributed to its anti-inflammatory, antioxidant, and metabolic regulatory effects. ISO exerts strong anti-inflammatory properties by upregulating the levels of secretory leukocyte peptidase inhibitor, suppressing Mincle/Syk/NF-κB signaling, and promoting M2 macrophage polarization, thereby reducing renal inflammation.^[46]^ This aligns with our mediation analysis, where WBC, NEUT, RDW, SII, and NLR levels partially explained the association of inflammatory makers with albuminuria risk. Additionally, ISO alleviates renal fibrosis and oxidative stress by inducing endogenous hydrogen sulfide (H_2_S) production, activating the Kelch-like ECH-associated protein 1/nuclear factor erythroid 2-related factor 2 signaling pathway, and reducing extracellular matrix deposition.^[17]^ It also enhances fatty acid oxidation via PGC-1α upregulation, mitigating mitochondrial dysfunction and restoring lipid metabolism in renal tubular cells.^[16]^ Further, ISO inhibits the activity of xanthine oxidase, reducing serum uric acid levels and preventing hyperuricemia-induced renal injury,^[15]^ while also suppressing PFKP-mediated glycolysis, a key driver of TGF-β1-induced renal fibrosis.^[47]^ Collectively, these findings suggest that ISO exerts renoprotective effects by modulating inflammation, oxidative stress, metabolism, and fibrosis-related pathways, which highlights its potential as a therapeutic candidate for CKD. Further interventional and longitudinal studies are needed to validate these findings.

This study had several strengths. Using NHANES, a nationally representative dataset, enhanced the generalizability of our findings. Also, the large sample size and rigorous statistical adjustments improved the robustness of our results. Moreover, using weighted logistic regression, RCS, and mediation analysis allowed for a comprehensive assessment of the relationship between dietary ISO intake, inflammation, and renal outcomes. Our analysis investigates inflammatory pathways as possible explanatory factors for the relationship between ISO intake and adverse renal outcomes.

However, our study also had several limitations. First, the cross-sectional design of this study prevented us from establishing causal relationships. Therefore, longitudinal or interventional studies are needed to confirm our findings. Second, dietary ISO intake was estimated based on 24-hour dietary recall data, which was subject to measurement errors and recall bias, potentially affecting the accuracy of intake assessments. Third, residual confounding could not be entirely ruled out because some unmeasured factors, such as detailed information on polyphenol metabolism or genetic variations, might influence the observed associations. Additionally, some laboratory biomarkers were measured only once in NHANES, without repeated assessments, resulting in bias. While acknowledging these limitations, our study provides preliminary epidemiological evidence that contributes to the growing body of research on ISO’s possible renal benefits. These observational findings highlight the need for rigorous prospective studies and mechanistic investigations to establish causal relationships.

## 5. Conclusions

Our study provides epidemiological evidence that higher dietary ISO intake is associated with a reduced risk of albuminuria, which is potentially mediated by its anti-inflammatory effects. This protective association may be attributed to the regulatory effects of ISO on inflammatory pathways and oxidative stress, as reflected by the reductions in WBC, NEUT, RDW, SII, and NLR. However, considering the cross-sectional nature of this study, further longitudinal and interventional studies are needed to establish causality and explore the therapeutic potential of ISO in CKD prevention and management.

## Acknowledgments

The authors are grateful to the National Health and Nutrition Examination Survey (NHANES) team for providing the data.

## Author contributions

**Data curation:** Chang Liu, Hanhan Kong.

**Formal analysis:** Guixia Li, Wujian Peng.

**Funding acquisition:** Wujian Peng, Peijia Liu.

**Methodology:** Guixia Li.

**Supervision:** Guixia Li, Peijia Liu.

**Validation:** Hanhan Kong, Guixia Li, Wujian Peng.

**Visualization:** Peijia Liu.

**Writing – original draft:** Chang Liu.

**Writing – review & editing:** Peijia Liu.
